# Study of Cytotoxic and Antibacterial Activity of Ag‐ and Mg‐Dual‐Doped ZnO Nanoparticles

**DOI:** 10.1002/open.202300093

**Published:** 2023-11-13

**Authors:** Aytan Azizi, Mohammad Ghasemirad, Bardia Mortezagholi, Emad Movahed, Seyed Sasan Aryanezhad, Ali Makiya, Hoda Ghodrati, Kamyar Nasiri

**Affiliations:** ^1^ Department of Endodontics Dental School Qazvin university of medical sciences shahid bahounar boulevard, P.O. Box: 3419759811 Qazvin Iran; ^2^ Department of Periodontics Faculty of Dentistry Rafsanjan University of Medical Sciences Khalije Fars Blvd., Pasdaran street, P.O. Box: 1946853314 Rafsanjan Iran; ^3^ Dental Research Center Faculty of Dentistry Islamic Azad University of Medical Sciences Shariati St, P.O. Box 19395-1495 Tehran Iran; ^4^ Oral and Maxillofacial Radiology, Private Practice Daroost street, P.O. Box 1944614581 Tehran Iran; ^5^ Student Research Committee, Faculty of Dentistry Mashhad University of Medical Science Mashhad Iran; ^6^ Department of Prosthodontics Shahid Beheshti University of Medical Sciences Daneshjoo Blvd, Velenjak, St., P.O. Box 1983969411 Tehran Iran; ^7^ Department of dentistry Islamic Azad University of Medical Sciences P.O. Box 19585-466 Tehran Iran

**Keywords:** Ag/Mg−ZnO, human breast cancer, *Streptococcus mutans*, Scanning Electron Microscopy, well-diffusion

## Abstract

A non‐laborious process for the fabrication of silver and magnesium dual doped zinc oxide nanoparticles (Ag/Mg−ZnO NP) is described. The wurtzite ZnO nano‐structures and the dual doped NP were analyzed by PXRD. SEM data showed the hexagonal morphology of our product, while the gathered anti‐bacterial outcomes towards *Streptococcus mutans* bacteria through micro‐dilution technic affirmed the enhanced performance of doped NP compared to the native ones. Furthermore, we gauged the toxic impacts of synthesized pure and Ag/Mg−ZnO NP against a breast cancer (MDA‐MB‐231) cell line through an MTT trial, which highlighted the superiority of the doped when compared to the native nanoparticles. In light of these comparisons, the applicability of Ag/Mg−ZnO NP in dental and medical science is proposed.

## Graphical abstract

A simple process for the fabrication of silver and magnesium dual doped Zinc oxide nanoparticles (Ag/Mg−ZnO NP) is described, which were studied for their anti‐bacterial effect towards *Streptococcus mutans* bacteria, as well as for their cytotoxic activity on a breast cancer (MDA‐MB‐231) cell line. In light of the collected toxicity and antibacterial data as a compared between the doped and native nanoparticles, the applicability of Ag/Mg−ZnO NP in dental and medical sciences is proposed.

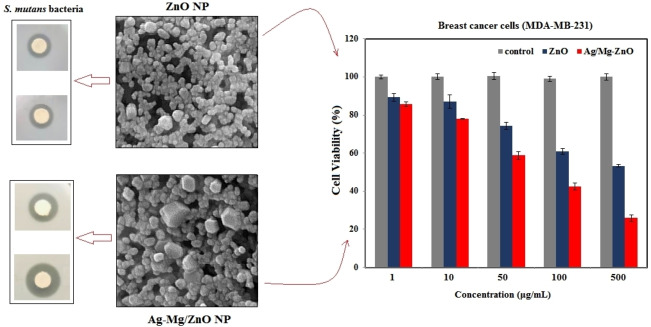

## Introduction

Zinc oxide NPs are biocompatible and safe nanoparticles that have been utilized as cosmetics, pharmaceutical carriers and medical fillers.[^1^, ^2^] The unique feature of ZnO NP include high chemical consistency, low dielectric steady, high catalytic performance, attraction of infrared and ultraviolet light, antibacterial feature. If anti‐tumor impacts of this NP are confirmed, this issue can be a main step in progressing cancer treatment techniques.[^3^, ^4^]

Dental caries is a microbial infectious disease that causes the decomposition and demolition of calcified dental tissue.^[5]^ The destruction procedure is the result of the carbohydrates activity of fermenting bacteria, acid generation and subsequent demineralization of dental tissues.^[6]^ Tooth decay is thought to be a microbial infective disease makes through several kinds of bacteria in mouth. One of the most major bacterial makes of dental caries is *Streptococcus mutans*.[^7^, ^8^]

The use of metals as antiseptic and antibacterial agents has been popular for a long time, but with the advent of modern and combined antibiotics that had a greater and faster effect and also considering the destructive side effects of metals and their compounds on living tissues, had forgotten this trait for a long time.[^9^, ^10^, ^11^] In recent years, the stunning progress of nanoscience and the subsequent discovery of new properties of nanoscale materials made researchers look for the key to the problem of the increasing resistance of pathogenic microorganisms in metal nanoparticles. Metal nanoparticles, in addition to having amazing properties and high potential in biomedical applications, do not have the destructive effects of metals and their ions in large quantities on human health.[^13^, ^14^, ^15^, ^16^, ^17^]

Zinc oxide nanoparticles show attractive antibacterial properties. The inherent toxicity of zinc oxide nanoparticles has caused them to have strong inhibitory effects against cancer cells and bacteria. Special emphasis has been placed on the mechanisms of bactericidal and bacterial growth arrest, focusing on their reactive oxygen species (ROS) production. ROS has caused cell wall damage due to local interaction of zinc oxide, increased membrane permeability, internalization of nanoparticle due to loss of proton motive force and absorption of toxic zinc ions in solution. Zinc oxide nanoparticles are toxic to cancer cells and cause the death of cancer cells through apoptosis. Induction of autophagy was positively correlated with their dissolution in lysosomes to release zinc ions, and zinc ions released from zinc nanoparticles were able to damage lysosomes and lead to disruption of autophagy and mitochondria.[^18^, ^19^, ^20^, ^21^]

Breast cancer, as common cancer for women, is caused by various parameters like oncogenes, growth factors and hormones.[^22^, ^23^] Chemotherapy methods based on alkylating agents and biological agents that are currently used to inhibit cancer cells are often unable to definitively treat cancer due to drug resistance or lack of detection of cancer cells. If it is possible to move the treatment methods in the direction of using natural substances and compounds derived from them with the least side effects, more efficient treatments can be achieved in this field.[^24^, ^25^] Nanotechnology is a new research science in the 21st century, which is derived from the distinctive properties of nanoparticles and their application in various fields.[^26^, ^27^] The usage of nanotechnology in medical usages, commonly mentioned as “nanomedicine”, following to provide a new of devices and therapeutic methods for therapy of diseases.[^28^, ^29^] By producing nanomedicines and targeting tumor cells, nanotechnology has been capable to take a major step in ameliorating the quality of the impacts of anti‐cancer drugs.[^30^, ^31^, ^32^, ^33^, ^34^] Zinc metal is involved in the structure of many enzymes and hormones and is used in various industries.^[35]^ Zinc oxide is one of the five known zinc compounds that have been widely used in medicine and nanotechnology.[^36^, ^37^] Plant extracts as a plant reducer can be used to produce green, easy and non‐toxic of nanoparticles.^[38]^ *Prosopis fracta* belongs to the Leguminosea family, which is native to arid and semi‐arid regions of Asia, Africa and America. This plant is a rich source of phenolic compounds, protein and oil, which phenolic compounds can play a potential reducer agent in synthesis of kind of nanoparticles.^[39]^ So, we presented an economical green synthesizing way for achieving pure and Ag/Mg−ZnO NP, and in continue, the antibacterial and cytotoxicity performance of pure and dual doped products are examined again *Streptococcus mutans* bacteria and MDA‐MB‐231 cell line.

## Results and Discussion

### PXRD

**Figure 1:**
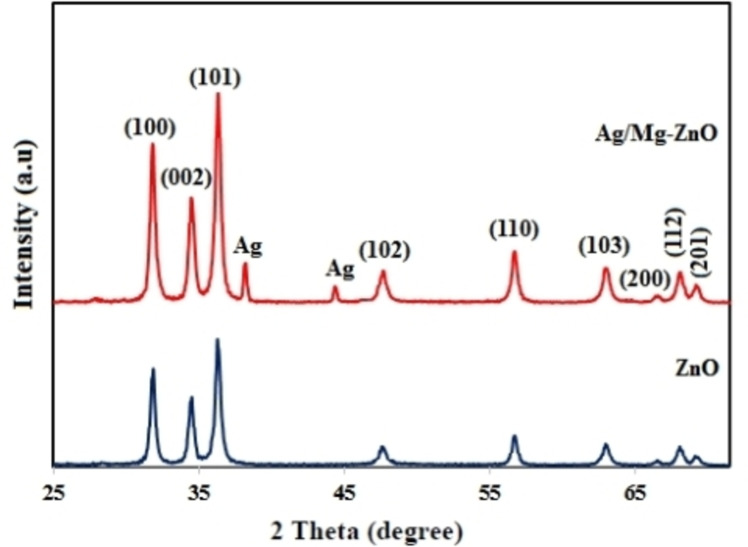
PXRD pattern of ZnO and Ag/Mg−ZnO nanoparticles.

Figure 1 illustrates the PXRD pattern of synthesized ZnO and Ag−Mg/ZnO NP through the employment of *P. fracta*. The perceived peaks for ZnO and Ag−Mg/ZnO NP were indexed to (100), (002), (101), (102), (110), (103), (200), (112), and (201) and seemed to correspond to the hexagonal form of ZnO (JCPDS‐36‐1451).^[37]^ Taking the data of Figure 1 in account, although the implication of silver in doped nanoparticles caused the appearance of Ag peaks at the PXRD pattern, yet the absence of detecting any other extra peaks signified the purity and supreme crystalline shape of synthesized product. We configured the crystalline size of our particles by the Debye‐Scherer formula in equation (D=Kλ/β cosθ; where D refers to the crystallite size of nanoparticles, K would be the shape factor, λ represents the wavelength of exerted radiation, β stands for the full width at half maxima (FWHM) in radians, and θ is the angle of diffraction). The full width at half maxima (FWHM) of XRD peak (101) was applied to Debye‐Scherrer formula^[37]^ for estimating the average crystallite size of 25.37 and 27.87 nm for ZnO and Ag−Mg/ZnO NP, respectively. In according to Figure 1, the impact of doped Ag and Mg metals into the crystalline network of ZnO nanoparticles on the extended crystalline size of doped NP may be accountable for the observed dissimilarity in the ionic radius of zinc atom (1.38 Å) in contrast to the doping of silver (1.26 Å) and magnesium (0.66 Å) atoms.

### FESEM and EDX

**Figure 2:**
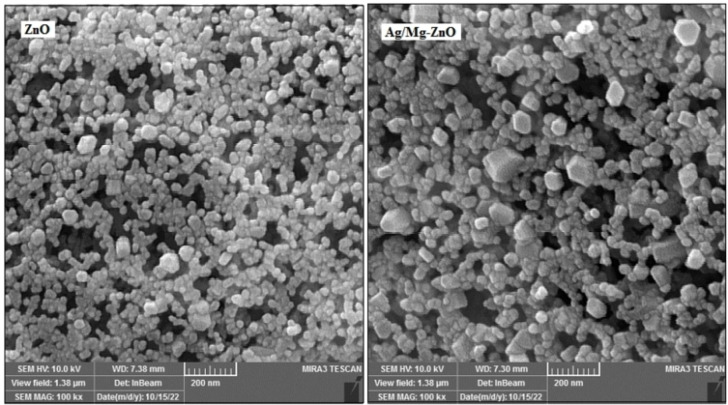
FESEM images of ZnO and Ag/Mg−ZnO nanoparticles.

**Figure 3:**
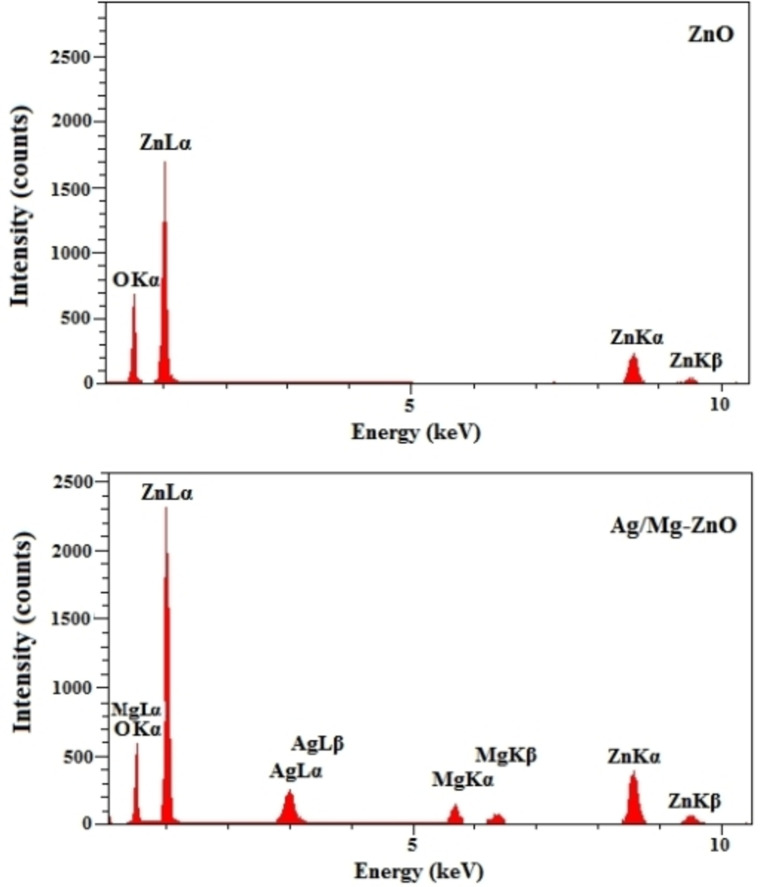
EDX profiles of ZnO and Ag/Mg−ZnO nanoparticles.

Figure 2 illustrates the FESEM images of ZnO and Ag/Mg−ZnO NP that were synthesized through the exploitation of *S. persica*, which confirm the close to spherical shape of ZnO particles along with a limited number of hexagonal ones throughout the doped nanoparticles. In addition, the images were prevailed an enlargement in the size of particles as an impact of doped Ag and Mg metals into the framework of ZnO. In accordance to Figure 3, the high‐purity content of ZnO and Ag/Mg−ZnO NP, as well as percentages of combination of O and Zn elements is 18.14 and 81.86 % in regards to the cases of ZnO; as well as it is 80.42, 17.24, 1.61, 0.73 % for Zn, O, Ag, and Mg elements of Ag−Mg/ZnO NP, was approved by the EDX outcomes.

### UV‐Vis

**Figure 4:**
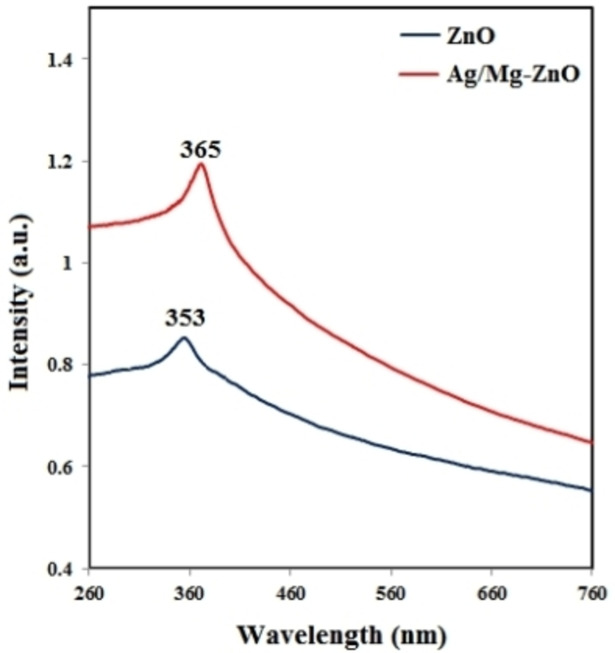
UV/Vis spectra of ZnO and Ag/Mg−ZnO nanoparticles.

The electronic constructions and their dynamics in atoms and molecules can be assessed through the analytical data of electron spectroscopy. Therefore, the electronic spectra of our ZnO and Ag/Mg−ZnO NP, synthesized through effects of *P. fracta*, were acquired and displayed in Figure 4, while their maximum wavelengths were perceived at the points of 353 and 365 nm, respectively. The effect of doped Ag and Mg within ZnO nanoparticles can effective on positioning and shifting of absorption spectra at higher wavelengths. This phenomenon is recognized as a red shift and apparently it is attributable to the altered amount of optical band gap, which is also known as an evident sign of reduced crystallization and quantum confinement impacts. An extension in the number of electrons in the course of doping Mg and Ag to ZnO can induce quantum constraints and even result in a red shift throughout the manner of optical absorption.^[17]^

### Antibacterial performance

**Figure 5:**
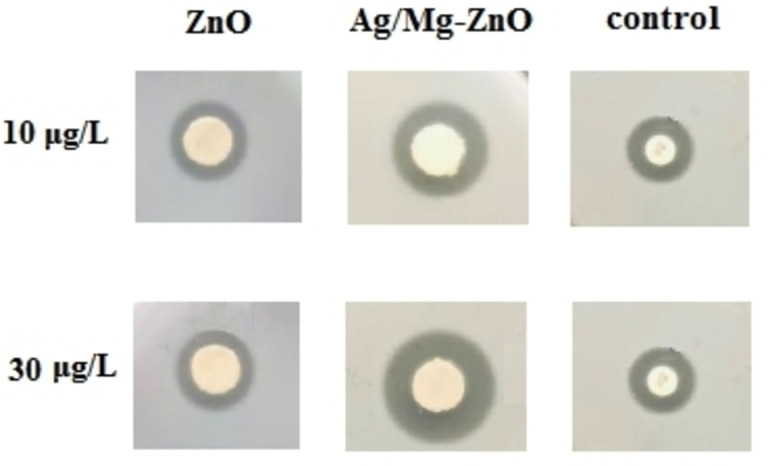
Antibacterial effects at 10 and 30 μg/L concentrations towards *S. mutans* bacteria.

The antibacterial efficacy of ZnO and Ag/Mg−ZnO NP in an experiment on *Streptococcus mutans* was determined through the presented outcomes of well diffusion technic in Figure 5. We perceived a noticeable level of inhibitory, which the outcomes of applying the dosage of NP (10 and 30 μg/L) presented in Table 1. The powerful antimicrobial influence of doped nanoparticles on *S. mutans* were reported and proved by a related study. As seen in Figure 5, silver and magnesium doped ZnO NP have higher impact compare to ZnO NP against *S. mutans*. The antibacterial behavior of synthesized NP towards bacteria are triggered by impairing the cell walls in order to distort the constructional proteins, make the enzymes inoperative, avert electron transport chains, deform nucleic acids (DNA), and assist the occurrence of oxidative stress by reactive oxygen species (ROS).[^40^, ^41^] Taking these facts into account, medical fields can exploit the benefits of exerting NP in antibacterial treatments. The usefulness of metal nanoparticles throughout medical implementations in terms of antimicrobial and anticancer functionalities was affirmed by some recent reports. Apparently, the biomedical applicability of metal oxide NP can be enhanced through boosting their features through the effects of doping processes on metal ions.^[42]^

**Table 1:** Evaluation of the antibacterial activity toward *S. mutans* bacteria.

	Diameter of Inhibition Zones (mm)		
Concentration	ZnO	Ag/Mg−ZnO	Control
10 μg/L	19±0.2	23±0.7	13±0.2
30 μg/L	21±0.18	25±0.3	13±0.2

### Cytotoxic performance

**Figure 6:**
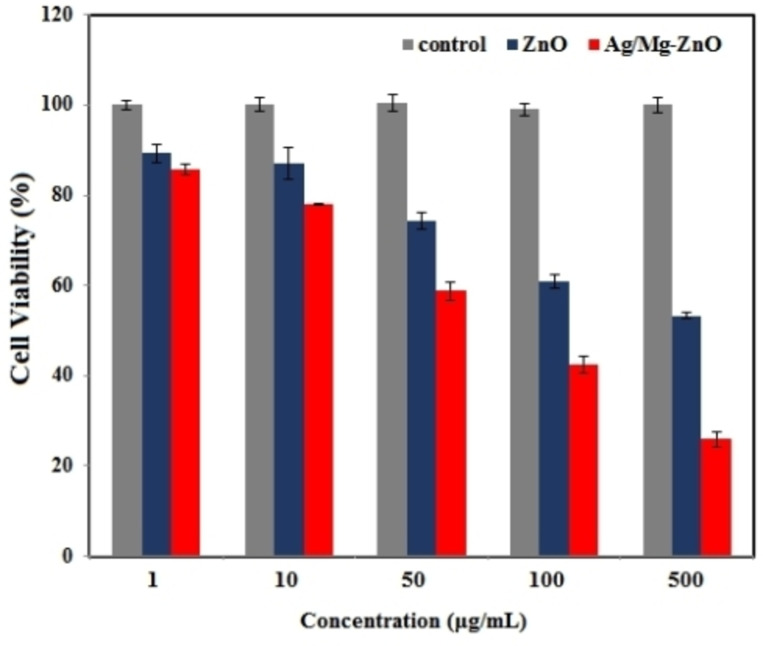
The cytotoxic activity of ZnO and Ag/Mg−ZnO nanoparticles.

This research implicated a section on examining the cytotoxic manner of ZnO and Ag/Mg−ZnO nanoparticles, synthesized through *P. fracta*, towards breast cancer cells (MDA‐MB‐231). The process included an MTT trial, which was began by the exposure of cells to varying volumes (1–500 μg/mL) of pure and dual doped ZnO nanoparticles for 24 h (Figure 6). The outcomes were indicative of an extension in cytotoxicity as a result of enlarging the applied concentration, which turned into a significant value at 500 μg/mL. We also perceived the superior cytotoxic influence of doped nanoparticles than the pure ones and realized the impact of doped silver and magnesium on raising the rate of induced cytotoxicity.

Numerous authors have reported the astonishing outcomes of exerting the green product of silver NP on cancer cells. For instance, the study of Swamy et al. succeeded in synthesizing silver nanoparticles by the exploiting the extracted leaf product of *Leptadenia reticulata*. In coordination to their MTT data, a dose‐reliant reduction was noted in the rate of cell viability and also, microscopic recordings exhibited a specific cellular morphological adjustment as a sign of morbid cells while the control was observed in a normal state.^[43]^ They also reported the detection of an extension in the rate of propidium iodide positive cells in the outcomes of maximum volume. Moreover, the anticancer performance of ZnO nanopowders was assessed through another research, which was manufactured through a solution combustion technic by exploiting the effects of bio fuels *Punica granatum* and *Tamarindus indica*. Taking the resulting viability values into account, arranged green ZnO nanopowders were applied at the highest amounts in our studies (100 μg/mL), whereas similar commercial product was capable of attaining an approximate cell viability of 66 %.^[44]^

## Conclusions

The synthesis of pure, and Ag and Mg dual doped ZnO NP was completed by involving the extract of *P. fracta* in a facile green procedure, which were confirmed to contain a hexagonal phase through the PXRD spectra. Based on the SEM mapping, the Ag and Mg were homogeneously distributed in ZnO while exhibiting high‐quality lattice fringes and lacking any sorts of crookedness. Furthermore, the cytotoxicity assessments were indicative of the superior toxic influence of doped NP towards breast cancer cells (MDA‐MB‐231) in contrast to the results of ZnO NP. It can conclusion that the doping of silver and magnesium metals lead to the more toxic impact for zinc oxide nanoparticles. The other section of our research involved the examination of our products capability in demonstrating anti‐bacterial behaviors towards *Streptococcus mutans* and the achievement of positive outcomes, which affirmed our offer in regards to suitability of synthesized doped NP for the diverse fields of dental health.

## Experimental Section

### Prepare of dual doped NP

**Figure 7:**
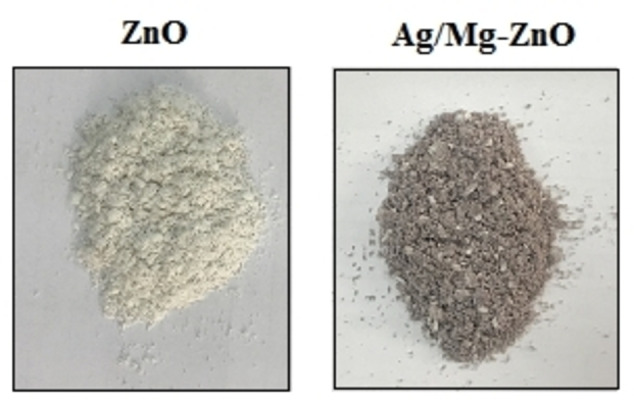
Images of obtained ZnO and Ag/Mg−ZnO nanoparticles using *P. fracta*.

The procedure was started by weighting and appending the *Prosopis fracta* leaves powder to distilled water (ratio 1 : 10) under shaking for 18 h at 150 rpm, which continued through filtering the resultant through a Whatman filter paper. Thereafter, we quantified 10 mL of extracted *P. fracta* to 50 mL by the inclusion of distilled water within two Erlenmeyer flasks to arrange a pure sample as well as a dual doped nanoparticles (Ag : Mg ratios is 2 : 1) sample, while the addition 0.583 g of zinc nitrate hexahydrate (0.02 M, Zn(NO_3_)_2_ ⋅ 6H_2_O, Merck) to each of the flasks was done as the next step. We appended the 0.012 g of magnesium nitrate hexahydrate (Mg(NO_3_)_2_ ⋅ 6H_2_O, Merck) and 0.06 g of silver nitrate (AgNO_3_, Merck) at the ratios of 1 : 2 on a heating stirrer at 80 °C for 2 h. Subsequent to drying the samples by an oven at 85 °C for 18 h, the procured raw product was put under calcination at 550 °C for 2 h. ZnO and Ag−Mg/ZnO NP are the labels given to the cases of pure, magnesium, and silver dual doped ZnO nanoparticles, respectively (Figure 7).

### Assessment of Inhibitory

This section refers to the experiment of a well‐diffusion trial on the inhibitory functionality of NP towards *Streptococcus mutans* bacteria, which was initiated through a streaking technic for inoculating the experimental bacteria into the sterile Petri plates that held 20 mL of Mueller‐Hinton. Subsequent to arranging the wells by sterile corn borer (6 mm), we proceeded by the inclusion of 200 μL of NP at 10 and 30 μg/L. The inoculated plates were refrigerated for 45 min to finish the adequate diffusion of NP, which was continued by being incubated at 37 °C for 24 h to assess state of bacteria. Gentamycin considered as a control. Ultimately, we configured the inhibition zone that surrounded each of the wells.

### Characterization

The findings were assembled through X‐ray diffractometer of PANalyticalX'Pert PRO MPD model, Field Emission Scanning Electron Microscopy or FESEM of MIRA3 TESCAN model, and UV‐Vis spectrometer of Rayleighuv‐2100 model. to configure the spectral response and defect states.

### Cytotoxic activity

#### Cell culture

The Pasteur Institute of Iran provided human breast cancer MDA‐MB‐231 cells for assessing the cytotoxic capability of these nanoparticles. Subsequent to thawing and thus culturing the cells, they were shifted to Falcon tubes to undergo a centrifugation process at 833 rpm for 9 min. Due to the involvement of a DMEM culture medium for cell culturing step, the microbial extension was required to be inhibited by the inclusion of 10 % fetal bovine serum (FBS), 100 μg/mL of streptomycin, and 100 international units/mL of penicillin to every culture medium. The incubation of culture medium at 5 % CO_2_ and 37 °C helped to proliferate and grow the cells.

#### MTT assay

A culturing process was done on Human breast cancer MDA‐MB‐231 cells by exerting an equipped high glucose DMEM with 10 % fetal bovine serum and 1 % penicillin/streptomycin solution within an incubator (37 °C, 5 % CO_2_) for the purpose of achieving cells count of 10000 in every well. Thereafter, we substituted the culture medium with 100 μL of DMEM composed of formulations in varying volumes (1, 10, 50, 100, and 500 μg/mL), which was seeded for one more round of 24 h while every case was incorporated with three duplications. Next to replacing the culture medium subsequent to 24 h, a fresh high glucose DMEM was also added and thereafter, 20 μL of 5 mg/mL 3‐(4, 5‐dimethylthiazol‐2‐yl)‐2, 5‐diphenyl tetrazolium bromide (MTT) solution was appended to every well for performing an extra step of incubation for 4 h. The obtained mixture from adding 100 μL of DMSO to every well was put under shaking for almost 15 min at ambient temperature to liquefy its formazan. Using a microplate reader, the absorbance of the samples was read at 570 nm, while the equation of VR=A/A_0_×100 % was employed to determine the rate of cell viability.

## Availability of data and materials

The present research results have not been published before. Data and Materials are all in the main text, figures and tables.

## Conflict of interests

The authors declare that they have no competing interests.

1

## Data Availability

The data that support the findings of this study are available from the corresponding author upon reasonable request.
